# Sustainability analysis of Finnish pre-schoolers’ diet based on targets of the EAT-*Lancet* reference diet

**DOI:** 10.1007/s00394-021-02672-3

**Published:** 2021-09-15

**Authors:** Sari Bäck, Essi Skaffari, Henna Vepsäläinen, Reetta Lehto, Elviira Lehto, Kaija Nissinen, Carola Ray, Jaakko Nevalainen, Eva Roos, Maijaliisa Erkkola, Liisa Korkalo

**Affiliations:** 1 Department of Food and Nutrition, University of Helsinki, P.O. Box 66, 00014 Helsinki, Finland; 2 Folkhälsan Research Center, Topeliuksenkatu 20, 00250 Helsinki, Finland; 3 Department of Teacher Education, University of Helsinki, P.O. Box 9, 00014 Helsinki, Finland; 4 School of Food and Agriculture, Seinäjoki University of Applied Sciences, P.O. Box 412, 60101 Seinäjoki, Finland; 5 Health Sciences/Faculty of Social Sciences, Tampere University, Arvo Ylpön katu 34, 33014 Tampere, Finland; 6 Department of Public Health, Clinicum, University of Helsinki, P.O. Box 63, 00014 Helsinki, Finland

**Keywords:** Planetary health diet, Sustainable diet, National Cancer Institute method, Children, Finland

## Abstract

**Purpose:**

The EAT-*Lancet* reference diet is a healthy plant-based diet produced within planetary boundaries. To inform the food system transformation, we compared Finnish pre-schoolers’ food consumption with the reference diet’s food group targets.

**Methods:**

Food record data for 3- to 6-year-old pre-schoolers were collected in the cross-sectional DAGIS survey. Ingredients of composite dishes were available in the data. In addition, we manually decomposed industrial products such as sausages and biscuits by estimating the shares of ingredients. We also estimated the consumption of added sugars and converted the consumption of dairy products into milk equivalents. We used usual intake modelling to estimate the mean consumption and the proportion of children who met the reference diet’s targets. We set the target amounts separately for 3- to 4-year-olds and 5- to 6-year-olds in grams by proportioning the published target amounts (assuming a 2500 kcal diet) to the children’s mean reported energy intake.

**Results:**

For both age groups (3- to 4-year-olds, *n* = 460; 5- to 6-year-olds, *n* = 402), the daily mean consumption of whole grains, vegetables, legumes, nuts, and unsaturated oils was below targets, whereas the consumption of red meat, dairy foods, tubers, and added sugars was above targets. The consumption of fruit and fish was in line with targets.

**Conclusion:**

To comply with the reference diet’s targets, major changes in the diets of Finnish children are needed. The key food groups targeted for higher consumption are whole grains and legumes and targeted for lower consumption red meat and dairy products.

**Supplementary Information:**

The online version contains supplementary material available at 10.1007/s00394-021-02672-3.

## Introduction

The global food system is a source of people’s well-being [1]. However, driven by the current diets, the food system generates adverse effects on both human health and the natural environment [2, 3]. As the world population is predicted to increase from about 7.7 billion in 2019 [4] to 9.7 billion by 2064 [5], these effects will intensify if food consumption patterns follow past trends linked to growing incomes [2]. The need and urgency of the transformation of the current food system towards a healthier and more sustainable direction are widely recognised [6–8].

The global food system is one of the most important drivers of environmental degradation [9, 10]. Operations to feed the world population are transgressing the planetary boundaries [11]. The food system accounts for approximately one-fourth of total greenhouse gas emissions, 60% of which derive from agriculture [12], the production of animal-based foods, such as beef, butter, cheese, and pork, having the highest greenhouse impact [13]. Over one-third of the terrestrial land is used for grazing and cultivation and taking land for agricultural use is primarily occurring in highly biodiverse ecosystems [14]. Environmental changes, in turn, may through decreased agricultural yields and higher food insecurity have consequences on people’s diets and health [15]. Today, food scarcity leaves nearly 690 million people undernourished [6].

In addition to the environmental burden, the current food system produces poor health outcomes, e.g. obesity and non-communicable diseases via unhealthy diets [7]. In the global food system, as populations become more affluent and urbanised, they tend to adopt Western-type diets high in energy, animal-based foods, and processed foods, which typically have elevated saturated fat, salt, and sugar, and low in fibre [16]. Suboptimal diets are ranked globally as a third leading risk factor of mortality for adults [17]. At the global level, the most highly ranking dietary risk factors among adults are high sodium intake and low consumption of whole grains, fruits, vegetables, nuts, and seeds [18]. Adopting healthy eating habits already in childhood is critical, as healthy diets are linked to long-term health benefits [19] and childhood diet tracks to adulthood [20].

To further the achievement of the United Nation’s Sustainable Development Goals and the Paris Climate Agreement, the EAT-*Lancet* Commission launched integrated food consumption and production targets to guide the transformation of food systems [8]. The EAT-*Lancet* Commission formulated a predominantly plant-based dietary pattern, called a reference diet (or a planetary health diet), which considered nutritional adequacy and is predicted to decrease premature deaths among adults by up to 24% [21]. The reference diet is proposed for healthy adults and children older than 2 years. The diet includes mass-based daily consumption targets for food groups with possible ranges, which are intended to inform national targets. The purpose of broad ranges for food consumption is to encourage and enable adaptation of the reference diet to local realities.

By setting targets, the EAT-*Lancet* Commission has enabled an examination of the gaps between current diets and the proposed sustainable diet. In Nordic countries, a difference between the EAT-*Lancet* targets and the current food consumption has been reported by Wood et al. [22] for adults in Denmark, Finland, Norway, and Sweden, and by Lassen et al. [23] for Danes aged 15–75 years. According to these studies, the consumption of red meat, dairy foods, and added sugars is excessive relative to the targets, while the consumption of vegetables, legumes, and nuts is below the targets. However, it is unclear how children’s food consumption relates to the EAT-*Lancet* targets.

This study aimed to provide an estimate of the gap between the reference diet and the current diet of pre-schoolers in Finland. This evidence is needed to identify the most pivotal action areas for the transformation of the food system to ensure better planetary health. To our knowledge, this is the first study to compare children’s food consumption against the EAT-*Lancet* targets. First, we adapted the EAT-*Lancet* targets for children based on their energy intake. Second, using recent food consumption data collected among Finnish pre-schoolers, we calculated the mean amount of consumed food in each food group of the reference diet. Third, we modelled the usual intake to ascertain the proportion of children meeting the targets in each food group of the reference diet. Finally, we discuss aspects in potential adaptation of the reference diet in the Finnish context.

## Methods

This study is a secondary analysis of the cross-sectional data collected in the DAGIS (Increased Health and Wellbeing in Preschools) study in 2015–2016. The sampling process was previously explained in detail [24] and is briefly described here. The study was conducted in eight municipalities in Southern and Western Finland. Eighty-six municipal pre-schools (56% of invited) consented to participate. All children in the groups for 3- to 6-year-olds (*n* = 3592) and their families were invited to participate by an invitation letter. Pre-schools having a too low participation rate (≤ 30% in all the groups for 3- to 6-year-olds) were excluded, leaving 66 participating pre-schools (43% of invited). The number of participants was 864 (29% of the invited children from the participating pre-schools). A parent or legal guardian of each participating child provided written informed consent. The University of Helsinki Ethical Review Board in the Humanities and Social and Behavioural Sciences reviewed the study protocol on 24 February 2015 and deemed it ethically acceptable (Statement 6/2015).

### Background data

Research personnel measured each child’s weight and height at pre-school and parents filled in the questionnaires querying their education level and whether their child followed a vegetarian diet containing any of the following: milk, fish, and egg, or not containing any of these [24]. The extended international body mass index cut-offs for thinness and overweight were applied [25].

### Food record data

Children’s food consumption was measured by food records collected both at home and at pre-schools. First, a 3-day food record data collection took place between September 2015 and April 2016, when exact dates to fill in food records (two weekdays and one weekend day) were assigned for each family. These dates were not always consecutive since the goal was to collect representative data covering all days of the week. Any changes to the assigned dates were negotiated between the family and the study group. Second, to detect seasonal contribution to the diet, a 2-day food record data collection was organized between June and September 2016. This time, the invitation was sent to the families that had consented to be contacted for additional data collection (*n* = 709, 20% of the invited children). The families chose 2 days (at least 1 day preferably being a weekday) for filling in food records during the week assigned to them by the study group. During the two collection periods, pre-school personnel recorded all foods and beverages that the child had consumed during pre-school hours on the same weekdays that the parents recorded all foods and beverages consumed outside pre-school hours.

Instructions for keeping food records were given to both the families and the pre-school personnel. The parents were equipped with food record instructions and sample pages by mail. The pre-school personnel were given both verbal and written instructions to fill in food records. These two parties used separate forms for keeping food records. They were instructed to record only the amounts actually consumed. The parents were asked to estimate portion size (i.e. volume, weight, size, and/or number of pieces) according to the validated [26] Children’s Food Picture Book [27] specifically developed for this purpose in the DAGIS Study, using household measures, food labelling, or weighing. In the case of composite dishes, parents were asked to record the ingredients and cooking method. For packed food products, the exact product or manufacturer name was required, including specific information about the product (e.g. fat content) when applicable. Pre-school personnel were given a food record form having predefined sections for breakfast, lunch, afternoon snack, and possible additional snacks. Also, different dishes, such as main courses, side dishes (potato, pasta, rice), and salad at lunch, each had predetermined rows. Personnel were asked to estimate the portion size using household measures or the same Children’s Food Picture Book [27] that the parents used. For packed food products served at the pre-schools (e.g. fat spreads, drinks), details were collected from the catering services.

The research assistants reviewed the food records upon their return, following a pre-determined procedure, and contacted the recorder for clarification if any food record data were incomplete or unclear. After the two data collection rounds, food record data for one or more complete days were available for 815 children. As the target age of participants was from 3 to 6 years, we excluded individual food record days when a child was aged under 3 or over 6 years. As a result, food record data from 1 to 5 days were available for 807 children (93% of the DAGIS survey sample) who formed the final sample for this paper.

Data were entered and processed using the dietary software AivoDiet 2.2.0.0 (Mashie FoodTech Solutions Finland Oy, Turku, Finland), including the Fineli food composition database (Release 16, 2013) [28] maintained by the Finnish Institute for Health and Welfare. To ensure calculations as precise as possible of the children’s food consumption, the following steps were taken by the study group. Food items and dishes appearing in the children’s food records but missing in the database were added and new recipes created. Recipes used in preschools’ catering services were added to the database when applicable. Five out of eight participating municipalities gave all of their recipes and one municipality some of its recipes. From the remaining two municipalities, no recipes were received, so recipes for their dishes were created based on the recipes used in the other municipalities.

### Disaggregation of food record data

The EAT-*Lancet* targets were provided for most of the foods in raw/dry form [8, 29]. Thus, we extracted the data from the dietary software in a format that disaggregates the recipes of mixed dishes into their ingredients. For some foods, mainly manufactured foods of milk, cereal, meat, or fat that did not have a recipe in the food composition database, we manually created approximate recipes (Supplementary Table S1). We estimated the relative shares of ingredients in weight using mainly product information available on Foodie online database [30], food manufacturers’ web pages, and/or wholesalers’ web pages. Owned by the S Group, the biggest Finnish retailer with 46% of the total market share, Foodie uploads the product information from S Group’s central system and/or the Synkka product information service [31] delivered by GS1 Finland Oy. Whenever practical, foods were converted to the same form used in the reference diet (Supplementary Table S2).

The EAT-*Lancet* Commission combined milk and dairy foods in the same food group informing the target amount in milk equivalents, which is a unit to measure the amount of liquid milk needed for producing a dairy product. Accordingly, we converted dairy foods to milk equivalents. As the EAT-*Lancet* Commission did not disclose details for calculating milk equivalents, we made our calculations using conversion factors (based on total solids) previously reported by Wood et al. [32]. We multiplied the amount of a dairy product by the relevant milk equivalent factor (milk 1.0, cream 2.7, cheese 5.0, and butter 6.5) and added the resulting product to the total amount of dairy foods in milk equivalents. The assignment of the factors for various dairy foods is shown in Supplementary Table S2.

As food composition databases do not provide explicit information about the amount of added sugars in the foods, we developed a calculation method, described earlier in Lehto et al. [33], to estimate the consumption of added sugars. Briefly, each food item was assigned to a food group, e.g. biscuits or yoghurts. To calculate the amount of added sugars in each food item, an average formula representing all foods in the group was applied to each food item. The relative amounts of naturally occurring and added sugars of the total sugar in a certain food were estimated using information from package labels, the Fineli food composition database [28], and commonly used recipes.

Finally, each food/ingredient in our data was manually classified into one of the reference diet’s food groups (Supplementary Table S2). Foods that are not included in the reference diet, and thus, were not included in our analyses, but were present in the children’s food records are listed in Supplementary Table S3.

### Conversion of the EAT-*Lancet* targets for pre-schoolers

The reference diet informs consumption targets both as a single target number and as a target range in each food group for adults having an energy intake of 2500 kcal/day. We used the NCI (National Cancer Institute) method, as described below, to calculate the mean energy intakes for two age groups (3- to 4-year-olds and 5- to 6-year-olds) (Table 1). The mean energy intake in the age group was divided by 2500 kcal/day, and the quotient resulting from the division was used as a multiplicative factor for adult targets to calculate targets for children in the two age groups. The targets calculated this way are shown in Tables 2 and 3.

**Table 1:** Characteristics of participants (*n* = 807) in the cross-sectional DAGIS study conducted in pre-schools in Finland

Characteristics	3- to 4-year-olds (*n* = 460)		5- to 6-year-olds (*n* = 402^a^)	
	Mean or percentage	25th/50th/75th percentiles	Mean or percentage	25th/50th/75th percentiles
Sex (%)				
Girls	48.3		46.8	
Boys	51.7		53.2	
Weight status^b^ (%) [25]				
Underweight	9.9		6.8	
Normal weight	80.5		79.2	
Overweight or obese	9.6		14.1	
Dietary intake (mean)				
Energy (MJ)	5.5	5.0/5.5/6.0	6.0	5.5/6.0/6.5
Energy (kcal)	1311	1185/1305/1428	1449	1325/1441/1563
Food records, number of days (%)				
1	0.7		1.0	
2	4.8		3.0	
3	69.1		63.9	
4	1.3		1.2	
5	24.1		30.8	
Vegetarian diets^c^ (%)				
Lacto-ovo vegetarian or pescovegetarian Highest educational level in the family^d^ (%)	0.7		0.5	
High school level or lower	20.2		21.4	
Bachelor’s degree or equivalent	44.1		41.3	
Master’s degree or higher	35.7		37.3	

^a^Of participants, 55 became 5 years of age during data collection, contributing data also to the age group 5- to 6-year-olds

^b^Data available from 426 participants for the 3- to 4-year-olds and from 384 participants for the 5- to 6-year-olds

^c^Data available from 452 participants for the 3- to 4-year-olds and from 397 participants for the 5- to 6-year-olds

^d^Data available from 456 participants for the 3- to 4-year-olds

**Table 2:** Daily food group consumption of 3- to 4-year-old^a^ Finnish children in the cross-sectional DAGIS study and comparison with the EAT-*Lancet* reference diet’s single target numbers

Food group	EAT-*Lancet* target (range), g/day^b^	Target, g/day	Mean consumption, g/day	Proportion of those who reached the target, %	25th percentile, g/day	50th percentile, g/day	75th percentile, g/day
Carbohydrate sources							
Whole grains	122	≥ 122	41	0	31	40	50
Tubers and starchy vegetables	26 (0–52)	≤ 26	69	0	55	67	81
Vegetables and fruits							
Vegetables	157 (105–315)	≥ 157	94	6	67	90	116
Fruits	105 (52–157)	≥ 105	132	64	91	124	165
Protein sources							
Dairy foods (in milk equivalents^c^)	131 (0–262)	≤ 131	645	0	494	635	783
Red meat	7 (0–15)	≤ 7	40	0	34	40	46
Beef and lamb	4 (0–7)	≤ 4	19	0	14	18	24
Pork	4 (0–7)	≤ 4	20	0	16	20	24
Chicken and other poultry	15 (0–30)	≤ 15	18	37	13	17	23
Eggs	7 (0–13)	≤ 7	12	15	8	11	14
Fish and seafood	15 (0–52)	≥ 15	18	66	14	17	22
Legumes	39 (0–79)	≥ 39	4	0	2	3	6
Nuts	26 (13–52)	≥ 26	1	0	0	1	2
Added fats							
Saturated fats	6.2 (0–6.2)	≤ 6.2	4	94	3	4	5
Unsaturated oils	21 (10–42)	≥ 21	11	0	9	11	13
Dairy fats (included in dairy foods)							
Added sugars	16 (0–16)	≤ 16	28	8	21	27	34

^a^Data from 460 participants including only the food record days when the child was 3–4 years old

^b^Assuming energy intake of 1311 kcal/day, which was the daily mean energy intake in the DAGIS study in the age group of 3- to 4-year-olds

^c^Includes dairy foods after conversion to milk equivalents (factors: milk 1.0, cream 2.7, cheese 5.0, butter 6.5 [32])

**Table 3:** Daily food group consumption of 5- to 6-year-old^a^ Finnish children in the cross-sectional DAGIS study and comparison with the EAT-*Lancet* reference diet’s single target numbers

Food group	EAT-*Lancet* target (range), g/day^b^	Target, g/day	Mean consumption, g/day	Proportion of those who reached the target, %	25th percentile, g/day	50th percentile g/day	75th percentile, g/day
Carbohydrate sources							
Whole grains	134	≥ 134	45	0	33	43	54
Tubers and starchy vegetables	29 (0–58)	≤ 29	77	1	61	75	91
Vegetables and fruits							
Vegetables	174 (116–348)	≥ 174	104	5	77	100	127
Fruits	116 (58–174)	≥ 116	146	63	97	136	184
Protein sources							
Dairy foods (in milk equivalents^**c**^)	145 (0–290)	≤ 145	682	0	521	669	826
Red meat	8 (0–16)	≤ 8	45	0	35	43	53
Beef and lamb	4 (0–8)	≤ 4	22	0	14	20	27
Pork	4 (0–8)	≤ 4	23	0	16	22	29
Chicken and other poultry	17 (0–34)	≤ 17	20	33	16	20	24
Eggs	8 (0–14)	≤ 8	12	24	8	11	15
Fish and seafood	16 (0–58)	≥ 16	19	61	13	18	24
Legumes	43 (0–87)	≥ 43	5	0	2	3	6
Nuts	29 (14–58)	≥ 29	2	0	0	1	2
Added fats							
Saturated fats	6.8 (0–6.8)	≤ 6.8	4	95	3	4	5
Unsaturated oils	23 (12–46)	≥ 23	13	0	11	13	15
Dairy fats (included in dairy foods)							
Added sugars	18 (0–18)	≤ 18	33	7	25	32	40

^a^Data from 402 participants including only the food record days when the child was 5–6 years old

^b^Assuming energy intake of 1449 kcal/day, which was the daily mean energy intake in the DAGIS study in the age group of 5- to 6-year-olds

^c^Includes dairy foods after conversion to milk equivalents (factors: milk 1.0, cream 2.7, cheese 5.0, butter 6.5 [32])

### Statistical analysis

The NCI method developed by the National Cancer Institute in the United States [34] was used for analyses. The short-term dietary data collection methods observe consumption with an error that can be corrected using the NCI method, which produces an estimate for long-term mean daily food consumption, i.e. usual food consumption. We estimated mean consumption for each food group with 25th, 50th, and 75th percentiles by the NCI method. We also used this method to estimate usual food consumption for the pre-schoolers and further to estimate the proportions of pre-schoolers meeting the food group consumption targets. Acknowledging that the EAT-*Lancet* Commission provided the single target number for each food group not to describe an exact diet but to enable calculations for overall diets, we used the single target numbers in our primary analysis. The reference diet encourages consumption of whole grains, vegetables, fruits, fish, legumes, nuts, and unsaturated oils over consumption of tubers, dairy foods, meat, eggs, saturated fat, and added sugars. Accordingly, in our primary analysis, we set the target so that the EAT-*Lancet* single target number was a minimum consumption amount for the former food groups and a maximum consumption amount for the latter food groups. Finally, we conducted a secondary analysis in which we estimated the proportions of children whose consumption was below, within, or above the EAT-*Lancet* target range for each food group.

We ran separate analyses for the two age groups. We calculated the age at each measurement day and split the data into two age groups based on current age. It should be noted that there was a 4- to 11-month gap between the first and second food records and some participants became 5 years of age (*n* = 55) during the data collection. Thus, these children contributed data to both age groups.

We used the model type ‘amount’ of the NCI method for frequently consumed food groups (i.e. whole grains, vegetables, fruits, dairy foods, unsaturated oils, and added sugars) and model type ‘corr’ for the rest of the food groups, consumed less frequently. We added current age (in years) and gender as dummy variables (parameter ‘covars_amt’) and considered Saturday and Sunday as the weekend days in all models. The usual intake modelling was carried out by SAS software 9.4 using the MIXTRAN and DISTRIB Macros.

## Results

### Participants

Girls accounted for just under half of participants in both age groups, the share of girls being lower in the older age group (Table 1). Of all children, 80% were of normal weight. Among all participants, 79% had at least one family member holding a bachelor’s degree or equivalent or a higher degree. Food records covered three or more days for 95% of all participants. The proportion of participants adhering to vegetarian diets was very small in both age groups.

### Food consumption compared with the EAT-*Lancet* targets

**Fig. 1:**
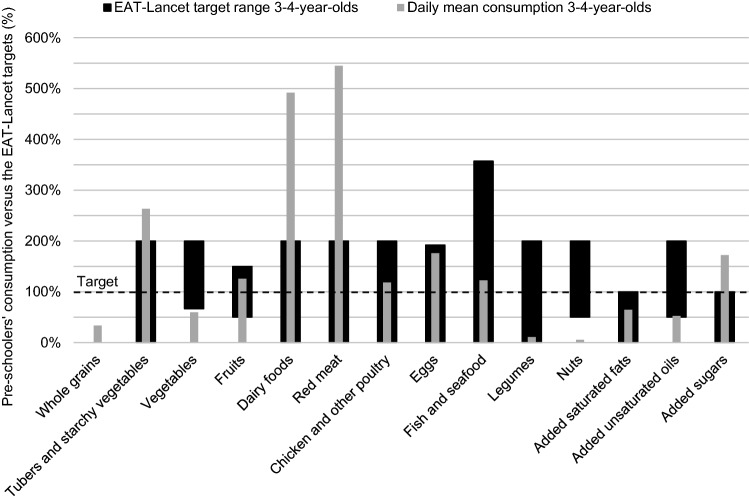
Diet gap between the daily mean consumption of 3- to 4-year-old Finnish pre-schoolers (*n* = 460, mean energy intake 1311 kcal/day) in the cross-sectional DAGIS study and the EAT-*Lancet* reference diet. The EAT-*Lancet* target level is indicated by the dashed line at 100%, and the thicker shaded bars represent the EAT-*Lancet* target ranges

**Fig. 2:**
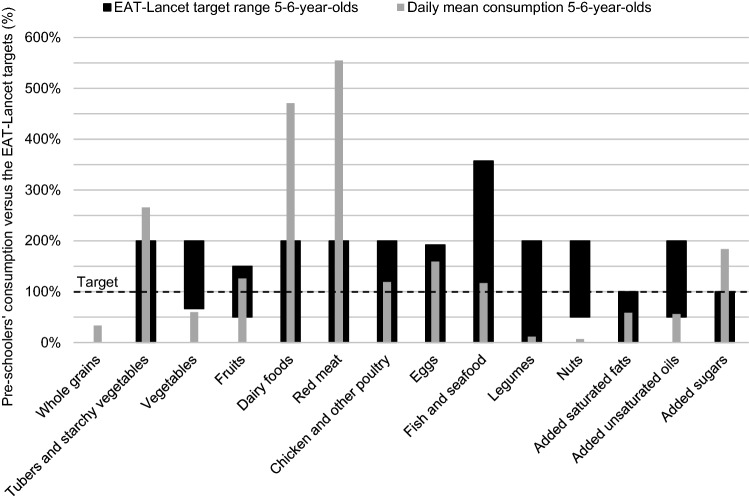
Diet gap between the daily mean consumption of 5- to 6-year-old Finnish pre-schoolers (*n* = 402, mean energy intake 1449 kcal/day) in the cross-sectional DAGIS study and the EAT-*Lancet* reference diet. The EAT-*Lancet* target level is indicated by the dashed line at 100%, and the thicker shaded bars represent the EAT-*Lancet* target ranges

The mean daily consumption of red meat (i.e. beef, lamb, and pork) and dairy foods exceeded the upper limit of the target range by more than twofold in both age groups (Tables 2, 3; Figs. 1, 2). Poultry and fish consumption were roughly in line with the target, with two thirds of children in the younger age group and close to two-thirds in the older age group achieving the target for fish consumption. Of children, 15% in the younger age group and a quarter in the older age group met the target for eggs. Children in both age groups consumed very few legumes and nuts.

The mean daily consumption of whole grains was only a third of the target in both age groups. In contrast, the mean consumption of tubers (i.e. potatoes) was more than double the target amount. Nearly two-thirds of the children consumed at least the target amount of fruit, while for vegetables the target amount was achieved by only approximately 5% of the children. The children did not attain the target for added unsaturated oils, but added saturated fats (i.e. combined palm oil, lard, and tallow) were roughly in line with the target. Lard and tallow were almost non-existent in the children’s diet, and thus, the food group of added saturated fats mainly comprised palm oil. Over 90% of children in both age groups consumed more added sugars than the upper limit of the target range.

The proportion of participants whose consumption was within the food group target range (Supplementary Tables S4 and S5) was higher in some food groups than the proportion of participants who met the food group target (Tables 2, 3). However, it should be noted that while the consumption of fish and legumes is encouraged in the reference diet their target range starts from zero. As a result, this type of comparison (Supplementary Tables S4 and S5) is hardly informative for these two food groups.

## Discussion

We compared the food consumption of Finnish pre-schoolers with the EAT-*Lancet* targets. The consumption of red meat and dairy foods was about fivefold the target. In contrast, the consumption of plant-based protein sources like legumes and nuts was very low. The consumption of whole grains should be triple, the consumption of unsaturated oils close to double, and the consumption of vegetables one and a half times higher to meet the target. Compared with the target, the consumption of tubers was over two and a half times higher and the consumption of added sugars nearly double. To our knowledge, this study provided the first analysis of children’s diets relative to the EAT-*Lancet* targets.

As the reference diet deviates markedly from the current food consumption of Finnish pre-schoolers, the adaptation of the diet to Finnish conditions considering prevailing food culture, food production, and climate is necessary. In contrast to the current food habits, the reference diet is rich in plant proteins and minimises the use of animal-source foods. Also, the reference diet does not include processing of foods, e.g. refining of grains. Consequently, the local adaptation would involve challenges such as the extent and the speed that transformations of the food system could be implemented in practice.

Refined grains are included in a vast range of products, e.g. bread, salty pastries, hamburgers, biscuits, cakes, and rice and pasta, which are widely consumed by Finnish pre-schoolers [35]. Also, some refined grains are typically included in whole-grain products. Within the European Union, labelling of whole-grain products is not harmonised, with policies varying between countries [36]. In Finland, products using the claim ‘whole grain’ are required to include a minimum of 50% whole grains of the cereal ingredients [37]. The use of refined grains, alone or mixed with whole grains, is preferred by the industry since refined grains have better qualities in baking by improving dough characteristics and allowing dough in bakery products to rise. An unrealistic change not only in the food habits but in the industry might be to swap all refined grains to whole grains, even though refined grains may be a nutritionally unnecessary part of the diet.

Globally, Nordic countries rank high in milk consumption per capita; Finland is the largest consumer of milk, and Sweden, Denmark, and Norway rank among the top 12 consumers [38]. In the northern climate, it has been more favourable to cultivate grass for livestock feed than to engage in other arable farming. Hence, cow’s milk production has become an important branch of industry in rural areas of Finland. The trend in liquid milk consumption has been downward for decades, while the trend in cheese and yoghurt consumption has been the opposite [39]. In Finland, whole milk is not commonly consumed by children, instead low-fat milk and skimmed milk are preferred [35]. Compared with the EAT-*Lancet* target, a substantially higher consumption of dairy products for pre-schoolers is suggested by Finnish food-based dietary guidelines, which state that daily consumption of 400 g of liquid milk products (e.g. milk, sour milk, yoghurt) and 10 g of cheese is enough to secure an adequate supply of calcium and iodine [40]. We found that pre-schoolers’ mean consumption of dairy products exceeded Finnish recommendations. These recommendations could, in fact, be the first goal to pursue.

Meat has a central role in the Finnish food system, in which beef and milk production are linked. Most of the beef comes to market as a by-product of milk production through male calves of dairy cows and cows that no longer produce milk. Typically, the diet of Finnish pre-schoolers contains dishes made from beef, pork, or poultry as well as processed meat such as sausages, frankfurters, and cold cuts [35]. In recent years, total meat consumption has been quite stable in Finland, as has beef consumption, while poultry consumption has continued to grow, and pork consumption has shrunk [39]. A significant reduction in meat consumption might have an impact on primary agriculture production in Finland [41]. Nonetheless, since meat is also imported to Finland the change may result in decreased imports rather than decreased domestic livestock production [42]. As consumption of meat is rooted in the food culture, transformation to the plant-forward diet may cause friction, which was recently demonstrated in an experimental study of sustainable eating at Finnish secondary schools, where pupils expressed resistance against vegetarian food [43]. However, students’ opposition to vegetarian school lunches might decrease over time [44].

Unlike in the EAT-*Lancet* reference diet, the role of legumes in the current food culture of Finland is minor, with a few exceptions such as the use of green peas in the traditional pea soup. The transformation to a plant-forward diet would entail introducing around ten times more legumes to the Finnish children’s diet as an alternative protein source to animal proteins. Jallinoja et al. [45] found that broadening of bean eating in Finland would require changes in people’s perceptions such as whether bean dishes are considered a part of the Finnish food culture, palatable, and easy to prepare. Consequently, legume consumption could be facilitated by developing tasty and attractive food products and legume dishes, learning skills to cook legumes in both home kitchens and professional kitchens, and introducing legume dishes to different consumer segments via mass catering. Increasing demand of dietary legumes in Finland would require investments in local product development, agricultural production, and food processing [41].

In the reference diet, tubers such as potatoes are optional foods, but in Finland the potato is an important crop and staple food. Over the past few decades, Finnish potato consumption has gradually diminished [39], while cereal side dishes, mainly rice and pasta, have gained popularity over the boiled potato. Nonetheless, rice, which is not cultivated in Finland and has a higher environmental impact than potato [12], could have a smaller role in future diets. Much of the evidence associating potatoes with negative health outcomes is linked to French fries, and there is less evidence for such outcomes for boiled and baked potatoes [46, 47]. A local adaptation of the reference diet, where boiled and baked potatoes and dishes have a somewhat larger role than suggested by the EAT-*Lancet* Commission, may be justifiable for Finland. In the Netherlands, the new food-based dietary guidelines already gave potato a target higher than that suggested by the EAT-*Lancet* target range [48].

The reference diet suggests a considerable consumption of nuts as a plant-based protein source. Due to their high fat and energy content, compared with the EAT-*Lancet* target, approximately half the amount of nuts, almonds, and seeds is recommended for pre-schoolers in Finland, and further, the consumption of seeds from oil plants is advised not to exceed 6–8 g/day, as they have a natural tendency to accumulate heavy metals [40]. In the whole diet, there should be some room for discretionary foods, although these are not recommended for frequent consumption and in high amounts. The reference diet excludes discretionary foods but includes a small amount of energy from added sugars (< 5 E%), while the recommended energy intake from added sugars (< 10 E%) is less stringent in the Nordic countries [49]. Among the Finnish pre-schoolers, the amount of added sugars consumed especially at home could be reduced [35].

Previously, Finnish Nutrition Recommendations, which are based on the Nordic Nutrition Recommendations (NNR), have been built on health-based evidence, and environmental sustainability of food consumption has been argued separately. An official forum where the possible adaptation of the EAT-*Lancet* reference diet in the Nordic region could be scrutinised is the NNR Committee, which is already working on incorporating sustainability aspects in the upcoming NNR 2022 on the request of the Nordic Council of Ministers [50].

In transforming food habits, the Finnish education system potentially plays a key role, as public pre-schools and schools nationwide serve meals and provide food education steered by the national law and policies. For each educational level, specific meal recommendations are provided that instruct on the nutritional quality of foods, menu design, and practical food education. Vegetarian dishes are recommended to be served at least once a week at pre-schools [51] and every day as an option in schools [52]. Additionally, the Finnish Government aims to increase the relative share of plant-based food in meals served by the public sector [53] and gives guidance for purchasers and catering services on responsible food procurement covering such aspects as environmental impact, social responsibility, and animal health [54].

### Strengths, challenges, and limitations

A strength of this study was a careful disaggregation of dishes and food products to raw ingredients to match the EAT-*Lancet* food groups. Another strength was a detailed collection of food record data, e.g. details of food products and recipes used by the pre-school catering services, and the use of a specifically designed picture book for children’s portion size estimation. However, food consumption data gathering and management, i.e. filling in the food record, followed by data entry and checking processes, and finally splitting and grouping the food items into analysis-ready food groups, usually entails some complexities and limitations.

Some methodological challenges occurred when translating children’s food consumption to the EAT-*Lancet* food groups. Manually assigning recipes for industrial products involved subjective decisions. As an example, because food manufacturers are not obliged to disclose the relative proportions of all ingredients in the product, we estimated missing proportions. Furthermore, generic food item codes (e.g. ‘a sausage’) were sometimes used in data entry, even though sausages come with varying product contents. In these cases, the ingredients were assigned to the item based on the content of a commonly consumed brand in a particular food group. Moreover, dark wheat flour is a speciality fibre-rich produce in Finland, which is used to manufacture ‘dark macaroni’. Technically, ‘dark macaroni’ is not a whole-grain food but was included in the whole-grain group in our analyses. The same was true for ‘dark rice’, the outer shell layer of which has been removed. The calculation of the amount of added sugars also involves using estimated factors, as information about added sugars in food products is missing in most cases from food composition databases. Furthermore, the milk equivalent amounts might vary depending on the method used to estimate them.

In Finland, preschool-based sampling reaches the target population well because the rate of participation in early childhood education and care is rather high. In 2016, it was 68% among children aged 1–6 years [55]. However, a limitation of the present study was that the study participation rate (29%) was quite low, and participants represented only two geographical regions in Finland. Also, the parents of participating children were quite highly educated, which may introduce some bias to the results.

### Prospects for future research

Transforming diets of Finnish children towards a more plant-based direction would require drastic changes in the current food consumption, some of which may be more favourable than others regarding the intake and bioavailability of nutrients. A study proposing the national adaptation of the reference diet in Denmark found that calcium and iron adequacy could be a major concern among children adopting the diet with decreased milk and meat content [23]. Accordingly, research on the possible consequences of adoption of a mostly plant-based diet, such as the reference diet, on dietary adequacy in children is warranted.

The reference diet was built based on health considerations, followed by modelling of the environmental sustainability of the integrated food production and consumption targets. However, a truly sustainable diet would also incorporate other aspects of sustainability such as economic and social factors. According to Hirvonen et al. [56], the reference diet would be unaffordable for nearly a quarter of the world’s population. Thus, actions would be required towards securing a food price-to-income ratio that allows shifting to healthy and sustainable diets. In Australia, a study [57] found that the price of a food basket would not increase if the EAT-*Lancet* targets were to be achieved, whereas in Finland such an analysis has not yet been conducted.

## Conclusions

To achieve a more sustainable diet and comply with the EAT-*Lancet* targets, Finnish pre-schoolers would need to consume more plant-based foods, i.e. legumes, nuts, and vegetables. The consumption of animal proteins, especially red meat, milk, and dairy products, in turn, would need to be decreased, as would the consumption of added sugars. Whole grains should replace the consumption of refined grains. The adoption of the reference diet would necessitate adapting the diet to reflect local realities such as the northern geographical location. Transforming the current diet closer to the reference diet would require drastic changes not only to the food habits of children and families but also at the food system level in Finland.

## Supplementary Information

Below is the link to the electronic supplementary material.

Supplementary file1 (PDF 291 KB)

## Data Availability

Researchers interested in the data from this study may contact principal investigator Eva Roos, eva.roos@folkhalsan.fi.

## Abbreviations

DAGIS: Increased Health and Wellbeing in Preschools
NCI: National Cancer Institute
NNR: Nordic Nutrition Recommendations
