# Supplementary material for: Sustainability analysis of Finnish pre-schoolers’ diet based on targets of the EAT-Lancet reference diet
Source: Eur J Nutr. 2021 Sep 15;61(2):717–28. doi: 10.1007/s00394-021-02672-3 (PMC8854143; doi:10.1007/s00394-021-02672-3)
Supplement: Supplementary file 1 — Supplementary file1 (PDF 291 KB) [file 394_2021_2672_MOESM1_ESM.pdf]

Supplementary material

Article title Sustainability analysis of Finnish pre-schoolers' diet based on targets of the EAT-*Lancet* reference diet

Journal name European Journal of Nutrition

Author names Sari Bäck, Essi Skaffari, Henna Vepsäläinen, Reetta Lehto, Elviira Lehto, Kaija Nissinen, Carola Ray, Jaakko Nevalainen, Eva Roos, Maijaliisa Erkkola and Liisa Korkalo[a]

[a] Corresponding author; University of Helsinki, Helsinki, Finland, e-mail: liisa.korkalo@helsinki.fi

Contents

Table S1 Examples of manually assigned recipes for the food products

Table S2 Foods and ingredients from the children's food records collected in the DAGIS study that were included in each food group of the EAT-*Lancet* reference diet

Table S3 Foods and ingredients from the children's food records collected in the DAGIS study that were excluded from the analyses

Table S4 Daily food group consumption of 3- to 4-year-old Finnish children in the cross-sectional DAGIS study compared with the EAT-*Lancet* reference diet's target ranges

Table S5 Daily food group consumption of 5- to 6-year-old Finnish children in the cross-sectional DAGIS study compared with the EAT-*Lancet* reference diet's target ranges

Table S1 Examples of manually assigned recipes for the food products

Food product	Recipe[a] (multipliers and food groups)
Rye bread, brand A	0.58 whole grains; 0.05 tubers and starchy vegetables
Rye bread, brand B	0.56 whole grains
Rye bread, brand C	0.45 whole grains
Rye crispbread	0.90 whole grains
Multigrain bread (incl. wheat and rye), brand A	0.03 whole grains
Multigrain bread (incl. wheat and oat), brand B	0.15 whole grains
Multigrain bread (incl. multigrain and seeds), brand C	0.05 whole grains; 0.12 nuts
Multigrain flour (incl. wheat and rye)	0.25 whole grains
Oat drink	0.1 whole grains
French fries, frozen	0.96 tubers and starchy vegetables; 0.04 unsaturated oils
Potato chips	0.70 tubers and starchy vegetables; 0.30 unsaturated oils
Instant mashed potato powder	0.89 tubers and starchy vegetables; 0.08 dairy foods
Tomato ketchup	0.60 vegetables
Jam from berries or fruits	0.40 fruits
Yoghurt incl. fruit or berry	0.85 dairy foods; 0.06 fruits
Ice cream	0.85 dairy foods
Pudding	0.86 dairy foods
Minced meat (mixed from pork and beef)	0.4 beef; 0.6 pork
Sausage, brand A	0.1 beef; 0.5 pork; 0.1 poultry
Sausage, brand B	0.1 beef; 0.2 pork; 0.2 poultry
Sausage, brand C	0.1 beef; 0.3 pork; 0.1 poultry
Ham, sliced	0.83 pork
Chicken fillet, marinated	0.72 poultry; 0.05 unsaturated oils
Pea soup, canned	0.06 pork; 0.4 legumes; 0.03 saturated fats (lard or tallow)
Soy drink, brand A	0.07 legumes
Soy drink, brand B	0.06 legumes
Cocoa biscuit with vanilla flavoured filling	0.04 dairy foods; 0.17 saturated fats (palm oil); 0.04 unsaturated oils
Cooking cream	0.3 dairy foods; 0.07 saturated fats (palm oil); 0.07 unsaturated oils
Fat spread, brand A	0.2 saturated fats (palm oil); 0.4 unsaturated oils
Fat spread, brand B	0.15 saturated fats (palm oil); 0.48 unsaturated oils
Fat spread, brand C	0.51 dairy foods; 0.24 unsaturated oils

[a] The recipe includes only the food groups of the reference diet that were used for preparation of foods. Each food group is preceded with the multiplier, e.g. 0.58 whole grains = 58% of the weight of the food product is assigned to the food group whole grains. The recipe comprises only the main ingredients of the food product and leaves out food groups not included in the reference diet, e.g. refined grains. Therefore, summing the factors in the recipe leads to a result of less than 1.

Table S2 Foods and ingredients from the children's food records collected in the DAGIS study that were included in each food group of the EAT-*Lancet* reference diet

EAT-*Lancet* reference diet's food group; form[a]	Foods and ingredients included in our analyses; form
Whole grains; raw	Whole-grain barley and rye, whole-grain wheat, dark wheat flour (fibre-rich non-whole grain) in dark macaroni, whole-grain rice, dark rice (outer shell layer removed), oat, millet, buckwheat, quinoa; mainly raw<br>Maize ears, maize kernels, popcorn; either raw or cooked<br>Oat from plant-based drinks and yoghurts
Tubers and starchy vegetables; raw	Mainly peeled potatoes; raw<br>French fries, potato chips, mashed potato powder, potato starch
Vegetables; raw/baked/boiled (includes dark green, orange and red, and other vegetables)	Root and leaf vegetables, fruit vegetables and other vegetables; raw<br>Ketchup, tomato crush and paste, pickles
Fruits; raw	Fruits and berries; raw (fresh)<br>Canned fruits, puree, jam, fruits and berries in dairy products, dried fruits
Dairy foods (in milk equivalents)	
Dairy products (milk equivalent factor 1.0[b])	Fat content below 15% (whole milk, skimmed milk, sour milk, kefir, yoghurt, Finnish cultured milk, curd, pudding, cottage cheese, cooking cream, vanilla sauce, ice cream, milk cocoa, milk powder)
Cream (milk equivalent factor 2.7[b])	Fat content 15% or higher: cream, creme fraiche, sour cream
Cheese (milk equivalent factor 5.0[b])	Fat content 15% or higher: edam, emmental, gouda, feta, mozzarella, cheddar, halloumi, cheese spread
Butter (milk equivalent factor 6.5[b])	Butter, includes butter in fat spreads (margarine and fat mixes) and shortenings
Beef and lamb; raw	Minced and whole meat of beef and lamb; mainly raw<br>Beef and lamb in processed meat: sausages, frankfurters, cold cuts, salami (incl. meat and ingredients comparable to meat)
Pork; raw	Minced and whole meat of pork; mainly raw<br>Pork in processed meat: sausages, frankfurters, cold cuts, salami (incl. meat and ingredients comparable to meat)
Chicken and other poultry; raw	Whole and minced meat of chicken, broiler and turkey; mainly raw<br>Chicken and turkey in processed meat: sausages, frankfurters, cold cuts, salami (incl. meat and ingredients comparable to meat)
Eggs; raw	Egg; mainly raw<br>Egg included in pastry, biscuits, cookies or mayonnaise

Fish and seafood; dry heat cooked	Mainly fish fillets of salmon, saithe and rainbow trout; raw<br>Some amounts of cooked or smoked fish<br>Canned tuna, fish fingers without batter
Legumes; raw	Mainly green peas; fresh<br>Dried or cooked peas, beans and lentils<br>Peas included in frozen vegetable mixes<br>Soy protein concentrates, soy flour, soy groat; dry/raw<br>Cooked soybean and tofu<br>Soy from plant-based drinks and yoghurts
Nuts; raw/roasted	Peanuts, peanut butter<br>Tree nuts<br>Smaller amounts of almonds and seeds, also seeds in bread
Saturated fats	Palm oil (for deep-frying)<br>Also palm oil included in fat products for baking, fat spreads (margarines and fat mixes), cooking creams, cookies, biscuits, savoury bakery products, popcorn, noodles<br>Chicken skin, lard
Unsaturated oils	Olive oil, rapeseed oil, sunflower oil, flaxseed oil<br>Also included in fat spreads (margarines and fat mixes), cooking creams, dressings, mayonnaise, liquid vegetable oil products, bread, cookies, biscuits
Dairy fats	Included in the group dairy foods
Added sugars	Sugar, honey<br>Also sugar added in berries, preserves, fruit and berry soups, conserves, smoothies, juice and soft drinks, breakfast cereal products, biscuits, cookies, cakes, pies, buns, doughnuts, pancakes, crêpes, porridge, rye/multigrain/wheat bread, milk-based drinks and desserts, yoghurt, ice cream, popsicles, oat- or soy-based drinks and products, almond drinks, sweets, chocolate, nuts, dried fruits, snacks, seasonings, spicy sauces, dressings

[a] From Supplement to: Willett W, Rockström J, Loken B et al (2019) Food in the Anthropocene: the EAT–Lancet Commission on healthy diets from sustainable food systems. Lancet. https://doi.org/10.1016/S0140-6736(18)31788-4. Accessed 7 Jan 2021

[b] From Wood A, Gordon LJ, Röös E et al (2019) Erratum: Nordic food systems for improved health and sustainability - baseline assessment to inform transformation. Stockholm Resilience Centre. https://www.stockholmresilience.org/download/18.8620dc61698d96b1901719c/1561013818461/Erratum_Nordic%20report_14-6-19.pdf. Accessed 7 Jan 2021

Table S3 Foods and ingredients from the children's food records collected in the DAGIS study that were excluded from the analyses

Foods and ingredients	
Refined grains	Non-whole grains in bread, biscuits, cereals, flours, semolina and liquorice, bran
	Non-whole grain macaroni and pasta (except dark macaroni made from fibre-rich non-whole grain dark wheat flour)
	White rice, rice starch
	Maize flour/starch/flakes
Game and offal	Elk, liver, blood, blood sausage
Other meat	Reindeer
Drinks[a]	Juice drinks and soups, 100% juices, soft drinks, water, mineral water, tea, coffee
Other foods[a]	Starch (except potato starch), salt, vinegar, seasonings, fish/vegetable/meat stock, yeast, mustard, sauce powders, soy sauce, meal and spicy sauces, coconut oil/flakes/milk, cocoa powder/butter/mass, food additives

[a] Other ingredients, except added sugars, were excluded from the analyses

Table S4 Daily food group consumption of 3- to 4-year-old[a] Finnish children in the cross-sectional DAGIS study compared with the EAT-*Lancet* reference diet's target ranges

Food group	EAT-*Lancet* target, g/day[b]	EAT-*Lancet* target range, g/day[b]	Proportion of those below the target range, %	Proportion of those within the target range, %	Proportion of those above the target range, %
Carbohydrate sources					
Whole grains	122	N/A	N/A	N/A	N/A
Tubers and starchy vegetables	26	0–52	N/A	20	80
Vegetables and fruits					
Vegetables	157	105–315	65	35	0
Fruits	105	52–157	4	67	29
Protein sources					
Dairy foods (in milk equivalents[c])	131	0–262	N/A	3	97
Red meat	7	0–15	N/A	0	100
Beef and lamb	4	0–7	N/A	2	98
Pork	4	0–7	N/A	1	99
Chicken and other poultry	15	0–30	N/A	92	8
Eggs	7	0–13	N/A	66	34
Fish and seafood	15	0–52	N/A	100	0
Legumes	39	0–79	N/A	100	0
Nuts	26	13–52	100	0	0
Added fats					
Saturated fats	6.2	0–6.2	N/A	94	6
Unsaturated oils	21	10–42	32	68	0
Dairy fats (included in dairy foods)					
Added sugars	16	0–16	N/A	8	92

N/A, not applicable
[a] Data from 460 participants including only the food record days when the child was 3–4 years old
[b] Assuming energy intake of 1311 kcal/day, which was the daily mean energy intake in the DAGIS study in the age group of 3- to 4-year-olds
[c] Includes dairy foods after conversion to milk equivalents (factors: milk 1.0, cream 2.7, cheese 5.0, butter 6.5 from Wood A, Gordon LJ, Röös E et al (2019) Erratum: Nordic food systems for improved health and sustainability - baseline assessment to inform transformation. Stockholm Resilience Centre. https://www.stockholmresilience.org/download/18.8620dc61698d96b1901719c/1561013818461/Erratum_Nordic%20report_14-6-19.pdf. Accessed 7 Jan 2021)

Table S5 Daily food group consumption of 5- to 6-year-old[a] Finnish children in the cross-sectional DAGIS study compared with the EAT-*Lancet* reference diet's target ranges

Food group	EAT-*Lancet* target, g/day[b]	EAT-*Lancet* target range, g/day[b]	Proportion of those below the target range, %	Proportion of those within the target range, %	Proportion of those above the target range, %
Carbohydrate sources					
Whole grains	134	N/A	N/A	N/A	N/A
Tubers and starchy vegetables	29	0–58	N/A	21	79
Vegetables and fruits					
Vegetables	174	116–348	66	34	0
Fruits	116	58–174	6	65	29
Protein sources					
Dairy foods (in milk equivalents[c])	145	0–290	N/A	3	97
Red meat	8	0–16	N/A	0	100
Beef and lamb	4	0–8	N/A	5	95
Pork	4	0–8	N/A	3	97
Chicken and other poultry	17	0–34	N/A	96	4
Eggs	8	0–14	N/A	69	31
Fish and seafood	16	0–58	N/A	100	0
Legumes	43	0–87	N/A	100	0
Nuts	29	14–58	100	0	0
Added fats					
Saturated fats	6.8	0–6.8	N/A	95	5
Unsaturated oils	23	12–46	39	61	0
Dairy fats (included in dairy foods)					
Added sugars	18	0–18	N/A	7	93

N/A, not applicable

[a] Data from 402 participants including only the food record days when the child was 5–6 years old

[b] Assuming energy intake of 1449 kcal/day, which was the daily mean energy intake in the DAGIS study in the age group of 5- to 6-year-olds

[c] Includes dairy foods after conversion to milk equivalents (factors: milk 1.0, cream 2.7, cheese 5.0, butter 6.5 from Wood A, Gordon LJ, Röös E et al (2019) Erratum: Nordic food systems for improved health and sustainability - baseline assessment to inform transformation. Stockholm Resilience Centre. https://www.stockholmresilience.org/download/18.8620dc61698d96b1901719c/1561013818461/Erratum_Nordic%20report_14-6-19.pdf. Accessed 7 Jan 2021)
